# Use of holmium laser for umbilical cord transection in a monoamniotic pregnancy threatened by an acardiac co-twin: a case report

**DOI:** 10.1186/s13256-022-03360-4

**Published:** 2022-04-06

**Authors:** Anouk M. van der Schot, Claire Jeltes, Joris van Drongelen, Mallory Woiski, Esther Sikkel, Frank P. H. A. Vandenbussche

**Affiliations:** 1 Department Obstetrics & Gynecology, Radboudumc/Amalia Children’s Hospital, Geert Grooteplein Zuid 10, Postbus 9101, 6500 HB Nijmegen, The Netherlands; 2 Department Obstetrics & Gynecology, Helios Klinikum Duisburg, Duisburg, Germany

**Keywords:** Ho:YAG laser, Umbilical cord, Occlusion and transection, Case report

## Abstract

**Background:**

Twin reversed arterial perfusion sequence is a rare complication of monochorionic multifetal pregnancies. In this syndrome, the acardiac twin has a nonfunctional heart, while the other twin, the pump twin, has normal development. The pump twin perfuses the acardiac twin and is therefore at risk for cardiac decompensation. In monoamniotic cases, the normal co-twin is also at risk of sudden death due to cord entanglement. Treatment consists of coagulation and transection of the acardiac’s umbilical cord. We report the first intrauterine use in pregnancy of a Ho:yttrium aluminum garnet laser to safely and successfully transect the umbilical cord after Nd:yttrium aluminum garnet coagulation.

**Case presentation:**

A 30-year-old Caucasian woman was referred to our fetal–maternal medicine unit at 9 weeks gestation with a monochorionic–monoamniotic twin pregnancy complicated by an acardiac twin. After counseling, she opted for an elective intervention to minimize the risks to the pump twin. At 16 weeks, fetoscopy was performed using a single 2-mm entry port. Through this port, a 1.0-mm fetoscope and a 0.365-mm laser fiber were introduced. Under fetoscopic sight and ultrasound (Doppler) guidance, the umbilical cord of the acardiac twin was first coagulated by laser energy using a Nd:yttrium aluminum garnet laser and then, using the same fiber, transected using a Ho:yttrium aluminum garnet laser. The patient underwent cesarean section at 38 weeks and delivered a healthy baby.

**Conclusions:**

We present the first report on intrauterine use of an Ho:yttrium aluminum garnet laser in human pregnancy. Ho:yttrium aluminum garnet laser energy can be successfully and safely used for umbilical cord transection and carries fewer risks than other methods of transection.

## Background

Twin reversed arterial perfusion (TRAP) sequence complicates 1% of monochorionic multifetal pregnancies [1]. In this syndrome, one of the fetuses, the so-called acardiac twin, has a nonfunctional heart, while the other, the so-called pump twin, has normal development. Around 25% of TRAP sequences occur in monoamniotic–monochorionic twin pregnancies [2]. In these monoamniotic cases, the pump twin faces three significant risks. First, there is a risk of congestive heart failure because the acardiac twin acts like a parasite, receiving blood from the pump fetus via a large arterio-arterial and returning it via a veno-venous anastomosis. Second, there is a substantial risk of fetal death by cord entanglement [3]. Finally, in all multifetal pregnancies, there is the risk of premature birth. Perinatal mortality of the pump twin exceeds 50%.

The outcome of TRAP can be improved by intrauterine intervention [3]. Treatment consists of interruption of blood flow through the acardiac twin by minimally invasive methods. In monoamniotic cases, cord transection should also be performed [4]. Cord transection has been described using a sole contact laser, a harmonic scalpel under ultrasonic guidance, scissors, and grasping forceps, but these procedures are complicated, require large-diameter entry points, and are not always successful [5–8].

This paper presents a case of TRAP sequence in a monoamniotic pregnancy that was successfully treated through a single 2-mm-diameter port. Umbilical cord occlusion was performed by using a neodymium-doped yttrium aluminum garnet (Nd:YAG) laser, and, using the same fiber, transection was performed using a holmium:YAG (Ho:YAG) laser.

## Case presentation

**Fig. 1:**
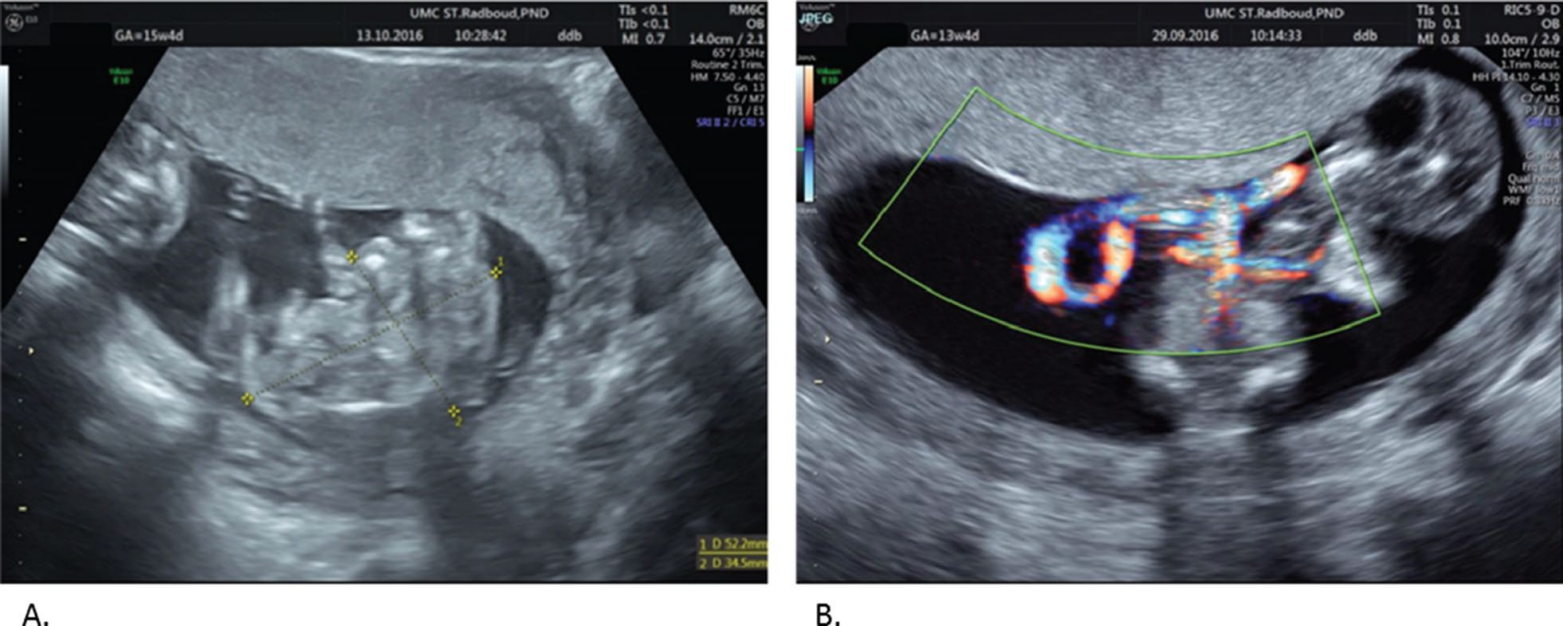
Ultrasonographic images of **A** acardiac twin and **B** cord entanglement, where blood flow is visualized

A 30-year-old primigravida, without relevant medical history, was referred at 9 weeks gestation with a monochorionic–monoamniotic twin pregnancy complicated by TRAP sequence. The first ultrasound revealed a structurally normal fetus accompanied by an amorphous fetus of similar size (Fig. 1A). Color Doppler imaging of the arcadiac’s umbilical cord showed a single umbilical artery with blood flow towards the acardiac twin.

At 11 weeks gestation, three possible options were presented to the patient and her partner: conservative management, elective fetoscopy with coagulation and transection of the acardiac’s twin ‘s umbilical cord at around 16 weeks, or intrauterine treatment only in the event of threatening congestive heart failure. The advantages and risks of these types of intervention were discussed in the Institutional Review Board (Ethical Committee), and approval was granted. We counseled the parents about the holmium laser’s first use in this case, with its anticipated advantages for transection. The patient opted for elective fetoscopy with cord occlusion and transection. From then on, we performed ultrasound weekly to define the optimal timing for intervention. Subsequent ultrasound examinations showed cord entanglement (Fig. 1B). No hydrops or congestive heart failure was seen.

### Procedure

At 16 weeks, fetoscopy was performed under peridural anesthesia. Amniocentesis was performed using an 18-gauge needle, and warm Ringer’s lactate was infused to create relative polyhydramnios and thereby an enlarged working environment. Using the Seldinger technique, a 2-mm-diameter 86Teflon cannula was inserted to guide a shaft containing a 1-mm fetoscope and a 0.365-mm laser fiber.

**Fig. 2:**
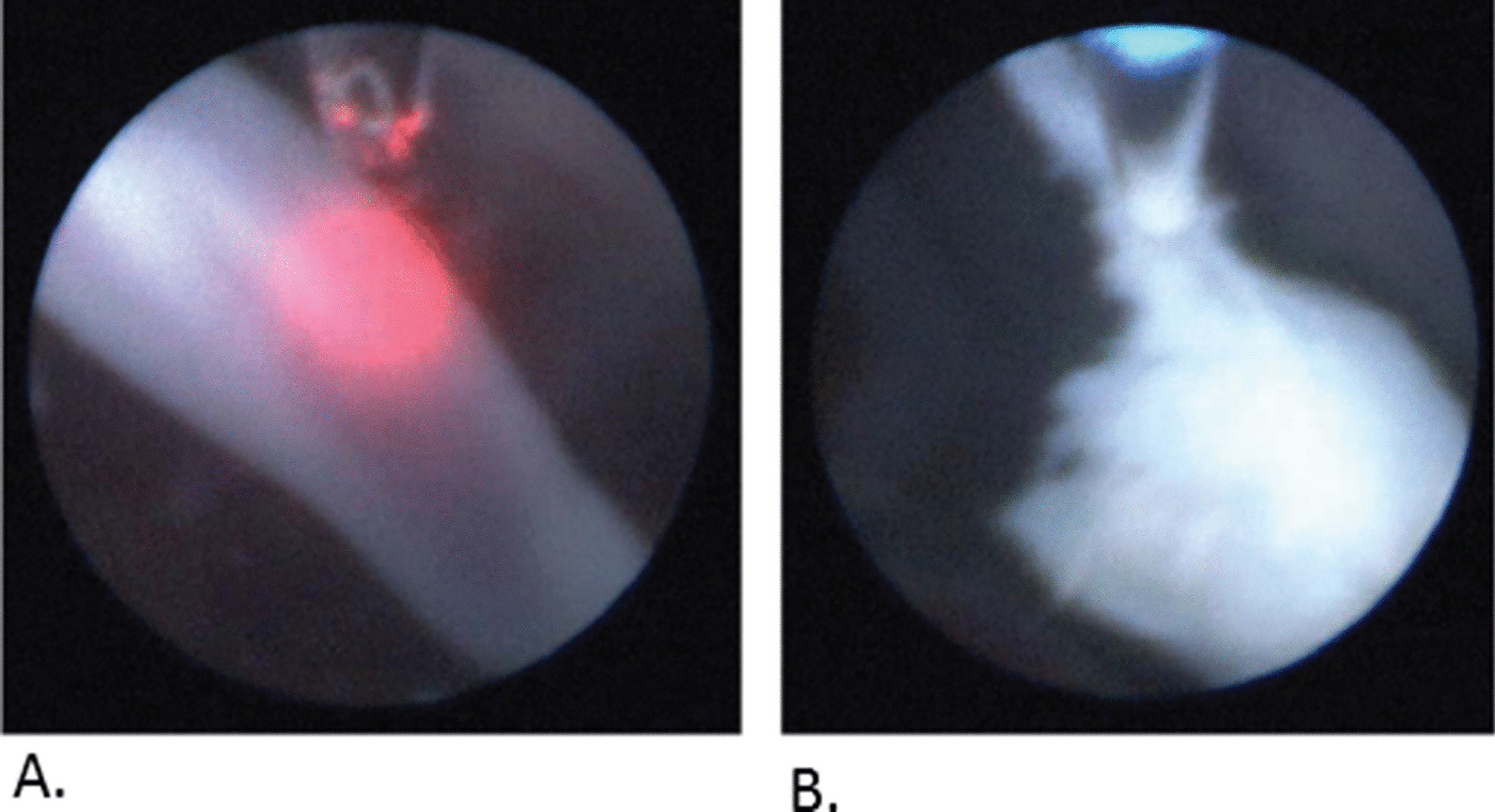
Fetoscopic images of **A** coagulation with Nd:YAG and **B** transection with Ho:YAG laser beam

Under fetoscopic sight and ultrasound guidance, the Wharton’s jelly and umbilical cord vessels of the acardiac twin were coagulated (Fig. 2A) using an Nd:YAG laser beam (50 W). Color and pulsed-wave Doppler ultrasonography was used to confirm the arrest of blood flow. After that, the Ho:YAG beam (0.5 J, 15 Hz) was used to transect the umbilical cord (Fig. 2B). The same laser fiber was used, only changing the setting from Nd:YAG to Ho:YAG.

**Fig. 3:**
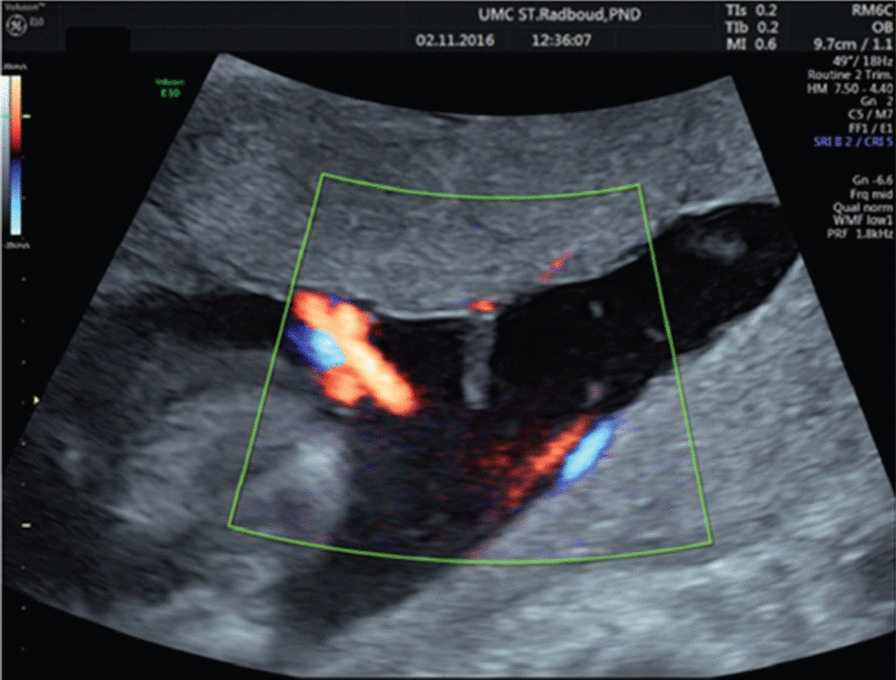
Postoperative ultrasonographic image of umbilical cord at placental insertion after cord transection

The Ho:YAG laser acts in direct contact with the umbilical cord and strictly cuts through the tissue without moving the cord. Due to its shallow penetration depth, there was no risk of unintendedly hitting other structures in the uterus (placenta, umbilical cord, etc.). Finally, extra fluid was drained. Postoperative ultrasound examination (Fig. 3) was regular, and the patient was discharged the same day. Six weeks later, she left the Netherlands and returned to her country of residence. At 38 weeks, the patient underwent cesarean section in her country and delivered a healthy male baby and the remains of a small acardiac twin. We had repeated email contact with the parents. They reported that the child was healthy and had completely normal development at the age of 4 years.

## Discussion and conclusions

This case report presents the first successful fetoscopic umbilical cord transection in a monochorionic–monoamniotic acardiac twin pregnancy using a Ho:YAG laser. The Ho:YAG laser is a solid-state, pulsed laser that emits light at 0.0021 mm (near the absorption peak of water), with a shallow penetration depth of 0.4 mm. This laser can cut or ablate tissue with slight charring and a thin necrosis zone [9, 10]. The laser’s active medium is the rare-earth element holmium in a YAG crystal.

Ho:YAG lasers have been effectively used and demonstrated to enable precise hemostatic cutting in many surgical specialties [11], and proved safe when used in pregnant patients. The sound intensities produced by this type of laser are shown not to be harmful to fetal hearing [12].

Umbilical cord occlusion and transection have been performed in various ways. Today, laser photocoagulation and bipolar electrocoagulation are the procedures used most frequently for cord occlusion, through a single 3-mm port [13]. Scissors, a harmonic scalpel, or a coaxial bipolar electrode (Versapoint) are used for cord transection [6, 8, 14]. Other, less invasive procedures include laser transection using variable laser sources. Middeldorp *et al*. were the first to report the use of a contact Nd:YAG laser to transect the umbilical cord [4].

In our case, we used a single 1-mm fetoscope and a single 0.365mm laser fiber to coagulate the cord with a Nd:YAG laser and subsequently transected the umbilical cord with a Ho:YAG laser. Using this method, we could use a single 2-mm entry point, considerably smaller than the 3.33-mm entry used in most other cases [4]. Using a single port with the smallest diameter carries less risk of premature rupture of membranes [15]. Also, direct contact and the absence of laser beams with Ho:YAG provide a minimal risk of collateral damage.

In conclusion, we report the first intrauterine use in pregnancy of a Ho:YAG laser to safely and successfully transect the umbilical cord after Nd:YAG coagulation. This minimally invasive single-port technique is easy to use and carries less risk than other methods of umbilical cord transection.

## Data Availability

Data sharing does not apply to this article as no datasets were generated or analyzed during the current study.

## Abbreviations

TRAP: Twin reversed arterial perfusion
Nd:YAG: Neodymium-doped yttrium aluminum garnet
Ho:YAG: Holmium-doped yttrium aluminum garnet
